# Concurrent Langerhans Cell Histiocytosis and Autoimmune Hepatitis: A Case and Review of the Literature

**DOI:** 10.7759/cureus.11808

**Published:** 2020-11-30

**Authors:** Ahmed Ahmed, Hasan Ali, Mark Galan, Jie-gen Jiang, Vivek Lingiah

**Affiliations:** 1 Internal Medicine, Rutgers University, Newark, USA; 2 Pathology, Rutgers University, Newark, USA; 3 Gastroenterology and Hepatology, Rutgers University, Newark, USA

**Keywords:** langerhans cell histiocytosis, autoimmune hepatitis, immunohistochemistry staining, acute gastrointestinal bleed

## Abstract

Autoimmune hepatitis (AIH) and Langerhans cell histiocytosis (LCH) are two independently rare disease processes that can have similar presentations. We present a unique, complex case that required a multidisciplinary approach to ultimately diagnose and treat the patient.

A 20-year-old male with no significant history presented with worsening jaundice, diffuse, pruritic rash, and abdominal pain over one month. On admission, the patient’s labs showed significantly elevated liver function tests (LFTs), eosinophilia, and anemia. The exam was notable for diffuse lymphadenopathy (LAD), hepatosplenomegaly, and a diffuse, non-blanching, morbilliform rash. Interdisciplinary workup was notable for positive anti-smooth muscle antibody (ASMA) and anti-neutrophilic antibody (ANA). A liver biopsy showed severe inflammation with interface activity, consistent with AIH. A lymph node (LN) biopsy showed findings consistent with LCH, including histiocyte clusters. He was started on high-dose steroids with LAD/LFT improvement; yet, his course was complicated by a gastrointestinal (GI) bleed requiring a hemicolectomy. The patient was transferred to a larger referral center where he continued to improve with steroids and was ultimately discharged.

This case was notable for an LN biopsy showing histiocyte clusters with reniform nuclei, nuclear grooves, and eosinophils with immunohistochemical stains positive for S-100, CD1a, fascin, langerin, CD45, and CD68, consistent with LCH. The resected colon showed atypical histiocyte proliferation positive for fascin, CD4, and CD68. Other findings, including elevated LFTs, ASMA, and a liver biopsy showing inflammation with interface activity, eosinophils, plasma cells, and characteristic fibrosis, supported a diagnosis of AIH. In either case, steroids were indicated.

## Introduction

AIH and LCH are two independently rare disease processes that can have similar presentations. LCH is a disease of uncertain etiology characterized by the abnormal proliferation of bone marrow-derived Langerhans cells that can affect any age group but has a predilection for children aged one to three years [1-2]. The incidence of LCH ranges from one to five cases per million per year, depending on age [2]. LCH predominantly affects the bone (77%), followed by the skin (39%), lymph nodes (19%), and the liver (16%) [2].

On the other hand, AIH is a chronic disorder that can present acutely and is characterized by hepatocellular necrosis and inflammation [3]. The incidence of AIH can be up to two per 100,000 population per year, making it relatively much more common than LCH [4-5]. The disease is known to affect females more than males, has no ethnic predilection, and can present at any age [4-7].

We hereby present a unique case of a 20-year-old man who was found to have biopsy-confirmed LCH concurrently with biopsy-confirmed AIH. Such a complex case required an interdisciplinary approach to ultimately diagnose and treat the patient. Of note, there have been no documented cases of simultaneous, clinically evident presentations of AIH and LCH. Therefore, this case and its review enhance the medical literature on such rare diseases. This article was previously presented as a meeting abstract at the 2019 American College of Gastroenterology Annual Scientific Meeting on October 27, 2019.

## Case presentation

A 20-year-old man with a history of severe acne complicated by neutropenia secondary to isotretinoin use and *Clostridium difficile *colitis secondary to tetracycline use initially presented to an outside hospital with worsening jaundice, diffuse pruritic rash, and abdominal pain for one month. He reported an unintentional 10-pound weight loss during this period and multiple clay-colored, soft bowel movements that worsened two weeks prior to presentation. At the outside hospital, ultrasound and magnetic resonance imaging (MRI) of the abdomen were done, which the patient endorsed, showed only a “thickened gallbladder.” He was discharged on ursodiol and diphenhydramine, which he was compliant with. However, he noticed worsening of his pruritic rash, which started as small, round lesions on his elbows that spread to his back and abdomen, at which time he was told by his gastroenterologist to discontinue the ursodiol. The rash continued to worsen, spreading over his entire body, which prompted his return to the hospital.

**Figure 1:**
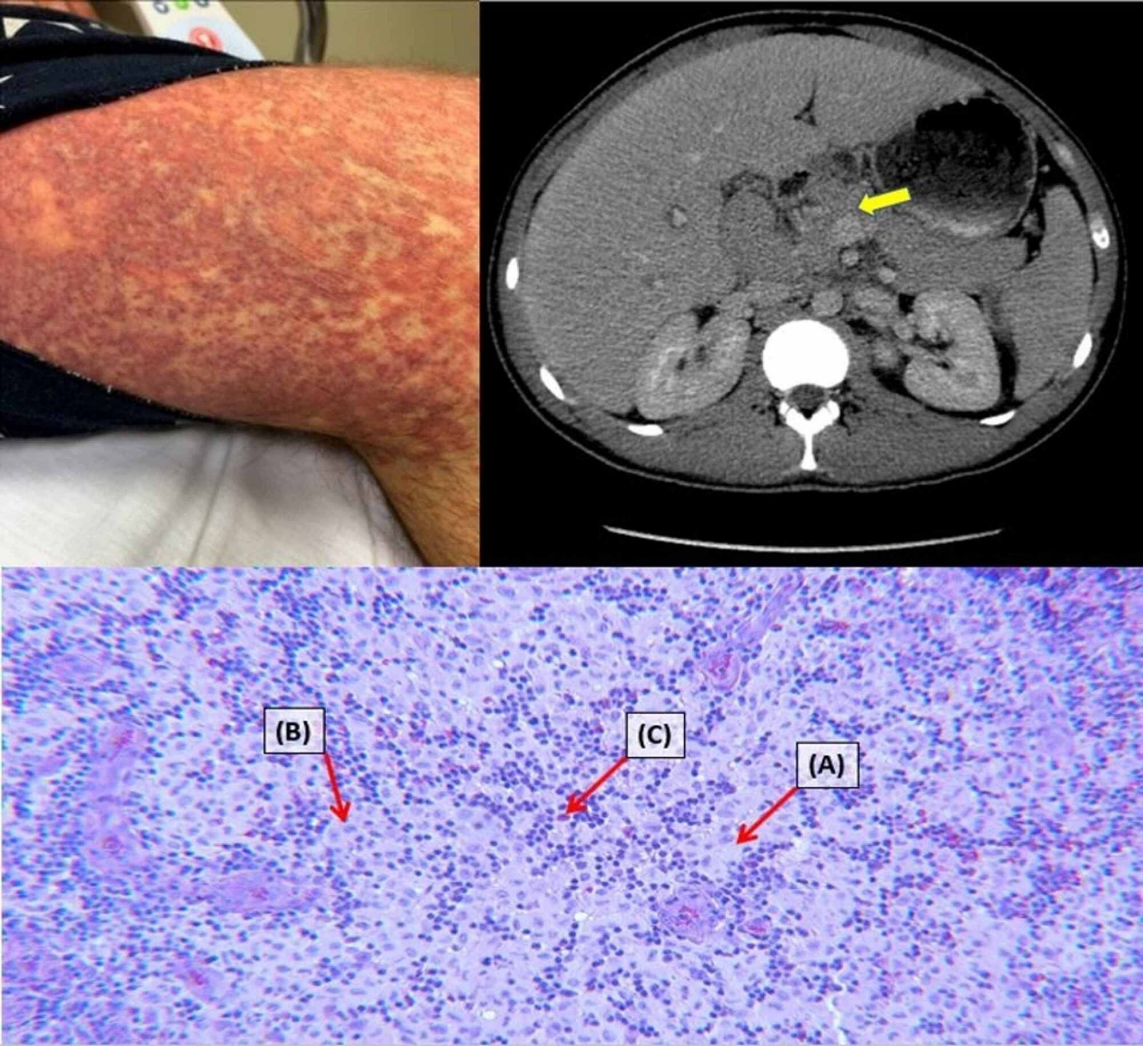
CT scan and rash presentation Top left image illustrates the patient's diffuse, non-blanching, morbilliform rash upon admission. The top right image portrays the computed tomography (CT) abdomen without contrast done on admission, which was notable for enlarged lymph nodes, including along the gastrohepatic ligament (arrow), as well as a heterogeneously enlarged liver. The bottom image is a higher magnification of the lymph node biopsy showing some key features consistent with a diagnosis of Langerhans cell histiocytosis: clusters of histiocytes with a reniform (kidney-shaped) nucleus and abundant, foamy cytoplasm (A), nuclear grooves (B), and the presence of inflammatory cells, including eosinophils (C).

Upon admission, the patient was alert and oriented, afebrile, tachycardic to 115 beats per minute, and mildly hypotensive (94/66). The exam was notable for bilateral lower extremity edema, jaundice, multiple enlarged, non-tender inguinal and cervical LAD, hepatosplenomegaly, and diffuse, non-blanching, flat, erythematous, morbilliform rash (Figure 1). He denied chest pain, dyspnea, nausea, vomiting, weakness, dysuria, or hematuria. He also denied any recent travel, sick contacts, nonsteroidal anti-inflammatory drugs (NSAID) use, alcohol use, drug use, risky sexual behavior, blood transfusions, or new medications aside from the discontinued ursodiol.

Lab work was significant for aspartate aminotransferase (AST) of 1572 U/L, alanine aminotransferase (ALT) of 1392 U/L, alkaline phosphatase (ALP) 148 IU/L, total bilirubin 14.2 mg/dL, direct bilirubin 9.9 mg/dL, protein 7.8 g/dL, albumin 1.9, sodium 129 mEq/L, bicarbonate 18 mEq/L, white blood cell (WBC) 6.5 with 25% eosinophils, hemoglobin 10.1 g/dL, and an international normalized ratio (INR) of 2.8. Imaging of the abdomen showed gastrohepatic ligament and porta-hepatic lymphadenopathy (LAD), enlarged spleen, and a heterogeneously enlarged liver (Figure 1). The patient was started on N-acetylcysteine (NAC) and vitamin K and had an infectious and rheumatological workup done for his acute liver failure. Of note, anti-smooth muscle antibody (ASMA) was positive at 46.2 and anti-neutrophilic antibody (ANA) was positive at 1:320. Viral studies, lymphoma panel, and other chronic liver disease autoantibodies were negative. His ceruloplasmin was normal and his ferritin level was only mildly elevated at 300 ng/mL.

**Figure 2:**
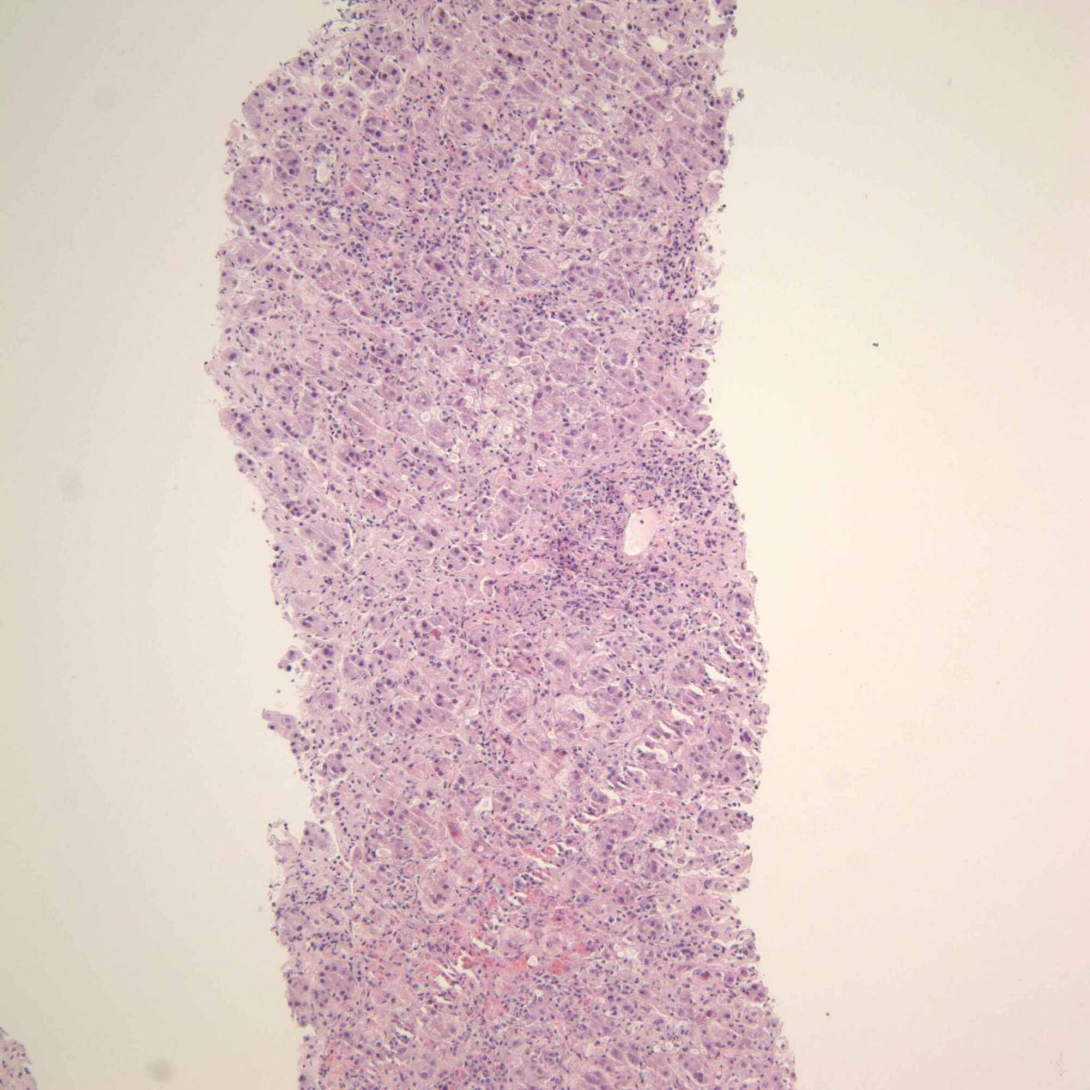
Liver biopsy Image from the liver biopsy (400x magnification) showing severe inflammation and confluent necrosis. It specifically highlights the inflammatory infiltrate, which contains numerous plasma cells and eosinophils with interface activity.

**Figure 3:**
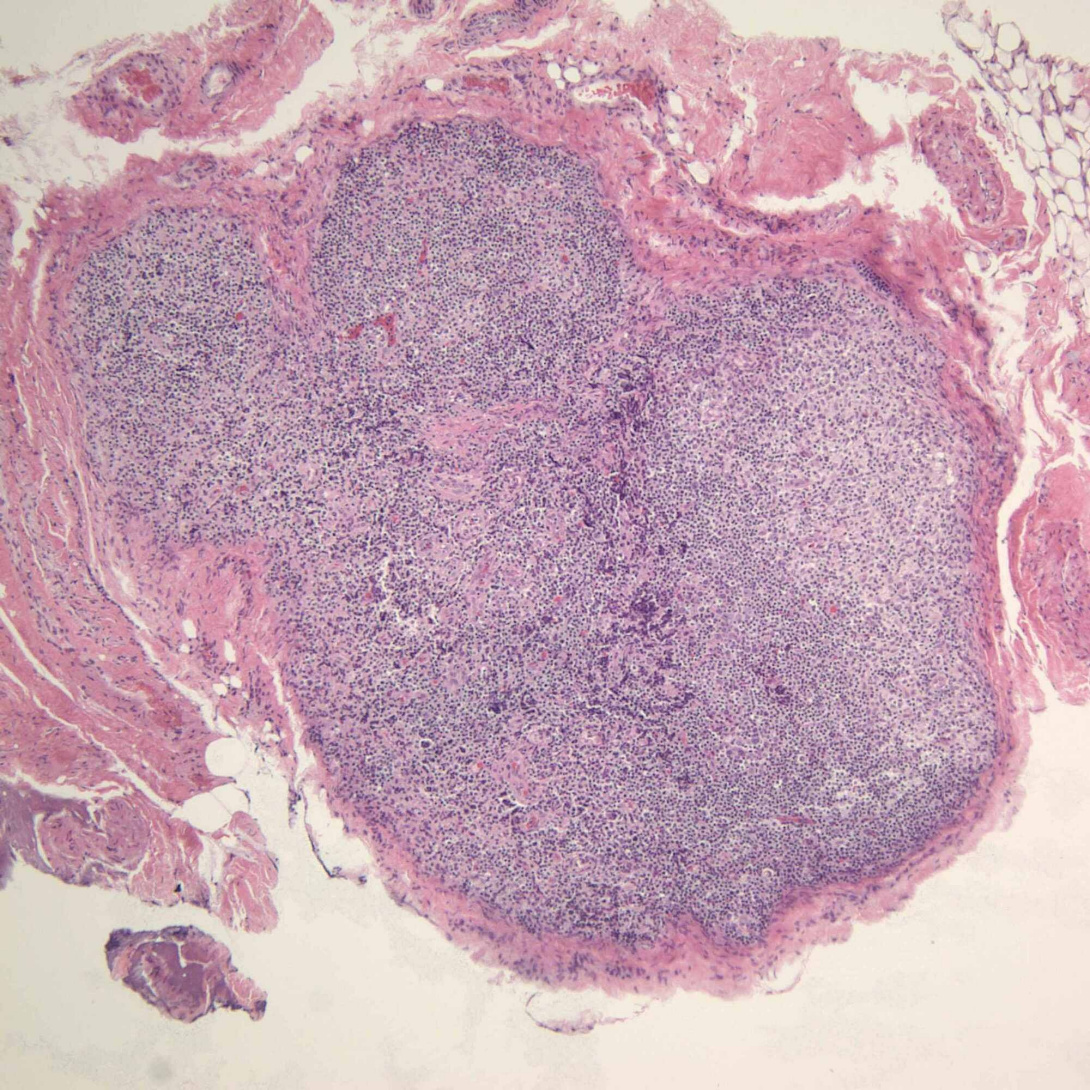
Lymph node biopsy H&E stain under 100x magnification of one of the patient’s inguinal lymph nodes showing clusters of histiocytes. H&E: hematoxylin and eosin

**Figure 4:**
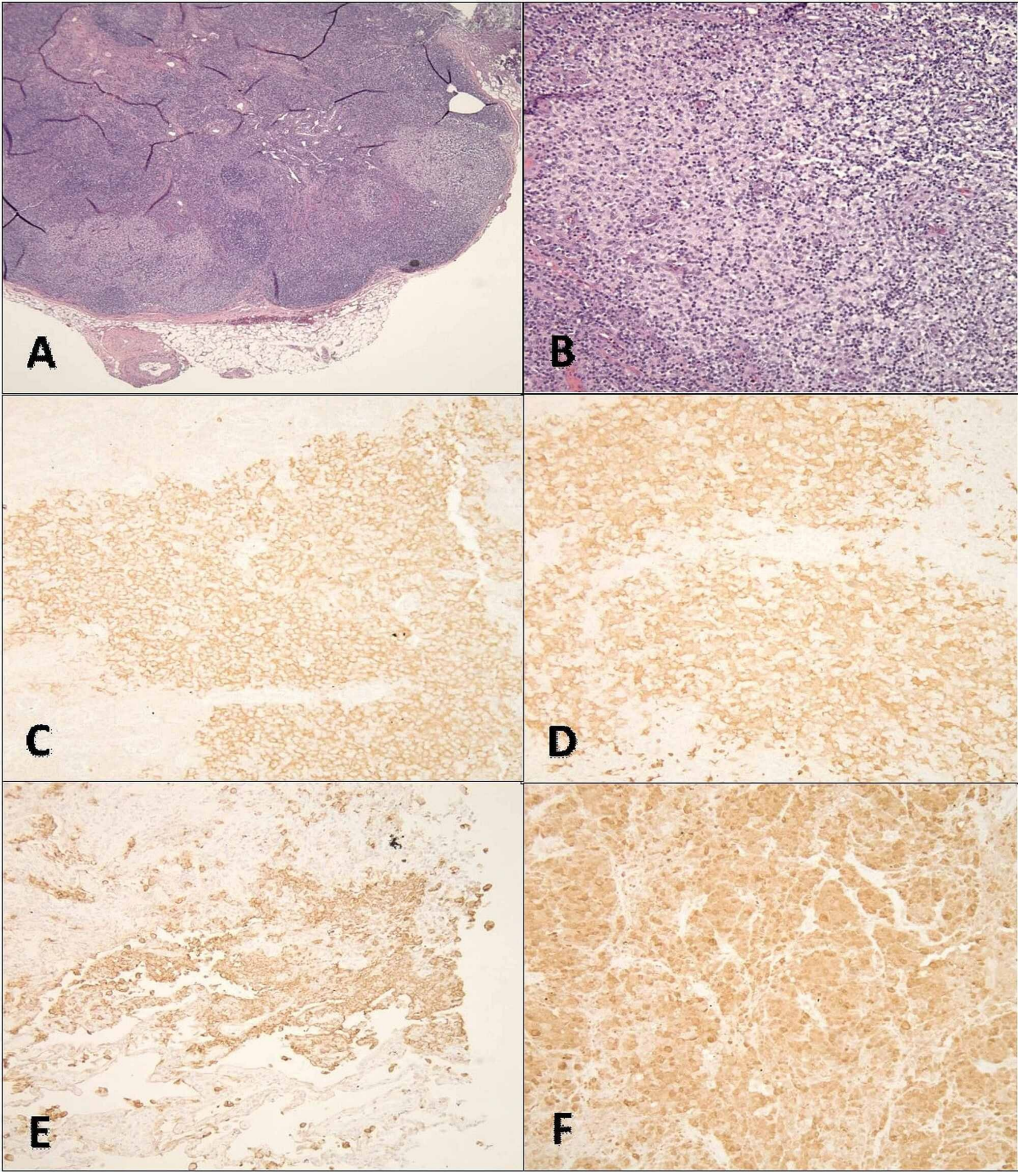
Immunohistochemical stains Image A demonstrates an H&E stain under 40x magnification of the inguinal lymph node with partial effacement of lymph node architecture by histiocytic proliferation (pale areas). Image B is a 200x view of histiocytic proliferation. Image C is a 200x view of an immunostain for CD1a, which highlights the Langerhans cells. Image D is a 200x view of an immunostain for S-100, which highlights the Langerhans cells. Image E is a 200x view of positive control for the immunostain CD1a for reference. Image F is a 200x view of positive control for the immunostain S-100 for reference. H&E: hematoxylin and eosin

A trans-jugular liver biopsy showed severe cholestasis and acute hepatitis with a marked mixed portal and lobular inflammatory infiltrate comprised of numerous eosinophils and plasma cells, as well as pericellular, periportal, and sinusoidal fibrosis with focal bridging (Figure 2). The patient was started on intravenous (IV) steroids for presumed AIH versus drug rash with eosinophilia and systemic symptoms (DRESS) syndrome. The patient also had an inguinal lymph node excisional biopsy, which showed histiocyte proliferation with nuclear grooves that were positive for S-100, CD-1a, fascin, langerin, CD-45, and CD-68, consistent with LCH (Figure 1, Figure 3, Figure 4). Computed tomography (CT) of the chest and a bone scan were done to workup LCH, both of which were negative for additional pulmonary or bone lesions. On further molecular testing, no BRAF V600E mutation, no IGH rearrangement, and no TCR rearrangement were detected.

After initiating glucocorticoids (methylprednisolone), the patient's liver enzymes began to improve, and his rash resolved. However, despite high-dose IV steroids, the patient’s mental status began to deteriorate. CT of the head was negative for any acute intracranial process, and he was noted to have elevated ammonia (112 µmol/L). He was started on lactulose enemas for presumed hepatic encephalopathy with a resolution of his symptoms. Three days later, the patient began to have several, large bloody bowel movements requiring the initiation of a massive transfusion protocol (MTP). He became hemodynamically unstable secondary to hemorrhagic shock, necessitating intubation and four vasopressive agents to maintain a mean arterial pressure (MAP) >60 mmHg. Abdominal angiography isolated the hemorrhage to the distal branch of the right ileocolic artery requiring emergent embolization by interventional radiology (IR), which was initially successful. However, three days after embolization, the patient had progressively worsening bloody bowel movements, again requiring MTP. Interventional radiology was unable to localize the source of bleeding on a repeat abdominal angiogram, necessitating an emergent right hemicolectomy with ileocolic anastomosis to control the bleed. Unfortunately, four days after his surgery, the patient experienced severe abdominal pain with an abdominal X-ray showing pneumo-peritoneum. An emergent exploratory laparotomy was performed, revealing anastomotic site leak with ascites, requiring abdominal washout and placement of an ileostomy.

The pathology results from the resection showed ulceration and chronic active inflammation extending transmurally to the serosal surface and involving the ileocecal valve, cecum, appendix, and ascending colon. It also showed atypical histiocyte/fibroblastic proliferation positive for fascin and CD68, but negative for CD1a, S-100, HHV-8, pancytokeratin, periodic acid-Schiff (PAS), Grocott methenamine silver (GMS), and acid-fast bacilli (AFB) staining. Cultures from the operating room grew vancomycin-resistant enterococcus (VRE) in the blood and *Citrobacter youngae* in the peritoneal fluid requiring IV antibiotics (daptomycin 6 mg/kg daily and meropenem 1 g every eight hours). The patient began to clinically improve, and a collective decision with the family was made to transfer the patient to a larger referral center where he continued to improve with steroids and was ultimately discharged with close follow-up.

## Discussion

AIH and LCH are two independently rare disease processes that can have similar clinical presentations, albeit with distinctive characteristics.

LCH is a disease of uncertain etiology that is characterized by the abnormal proliferation of bone marrow-derived Langerhans cells, which are myeloid progenitor cells or committed dendritic cell precursors [1]. Langerhans cells are a type of antigen-presenting cells (APC) with tropism for the skin and mucosa. Epidermal Langerhans cells find their origin in yolk-sac progenitors and fetal liver-derived monocytes that migrate to the skin before birth. Once activated, they migrate via chemokine receptor-dependent translocation to local draining lymph nodes, where they interact with T cells [1,8]. As APCs, they help trigger T cell-mediated immune response to an engulfed antigen [8]. Thereafter, they are cleared via apoptosis, among other mechanisms [1].

LCH has been diagnosed in all age groups but is most common in children aged 1-3. In adults, the incidence can range from 1-2 cases per million, whereas in the pediatric population, it is up to 5 cases per million [2]. LCH is known to mainly affect the bone (77%), followed by skin (39%) and lymph nodes (19%), and then the liver (16%) [2]. Gastrointestinal (GI) involvement is rare with only a few cases reported and occurs mainly in a multisystem presentation [9].

LCH often presents a diagnostic challenge since it is a rare disease that can affect multiple organ systems. Moreover, it is commonly confused with lymphomas or other common solid tumors [10]. LCH must be distinguished histologically and immunophenotypically from various other neoplasms and histiocytic syndromes, thus requiring a tissue sample in addition to the clinical context for a diagnosis. Biopsies of involved tissue typically portray heterogeneous collections of histiocytes with inflammatory cells including eosinophils and neutrophils [10]. Langerhan cells morphologically present as large oval mononuclear cells with few cytoplasmic vacuoles and an abundance of eosinophilic, foamy cytoplasm. The nucleus often has a “coffee bean” appearance, as it has fine chromatin with thin, grooved nuclear membranes [10]. Immunohistochemically, like dermal Langerhans cells, LCH cells express markers CD1a, S-100, and langerin.

This case was notable for a lymph node biopsy demonstrating key features such as clusters of histiocytes with a reniform or “coffee bean” shaped nucleus that had nuclear grooves and abundant, foamy cytoplasm, as well as the presence of eosinophils and other inflammatory cells (Figure 1, Figure 3). Moreover, the biopsy specimen stained positive for S-100, CD1a, fascin, langerin, CD45, and CD68. Such histological and immunohistochemical findings were consistent with a diagnosis of LCH.

The literature varies on whether LCH should be classified as an autoimmune, immunoregulatory, or neoplastic disorder, which ultimately affects proposed treatment therapies. However, studies have suggested a neoplastic etiology rather than a reactive process due to the discovery of somatic mutations isolated in most cases [11]. Activating mutations in the mitogen-activated protein kinase (MAPK) transduction pathway, which are essential in activating transcription of genes for several cellular processes, have been observed in molecular studies of many LCH lesions. Specifically, several studies have demonstrated how pathological Langerhans cells carry a BRAF V600 mutation in CD34+ stem cells and mature myeloid dendritic cells of LCH patients, which constitutively activates the MAPK pathway [12]. Although in this case, the patient was negative for BRAF V600, other activating mutations of BRAF have also been implicated in LCH lesions, as have mutations in other kinases in the MAPK pathway and other activating somatic mutations in upstream receptor tyrosine kinase genes such as ERBB3, NRAS, and KRAS [1]. It has been proposed that the state of differentiation of the precursor cells in which these activating mutations occur can be correlated with the severity of the disease. Mutations that occur in less-differentiated, pluripotent cells tend to give rise to more aggressive, disseminated, and fatal manifestations of LCH than do mutations in more committed, further-differentiated precursors [1]. Moreover, the literature has been in constant debate in terms of the therapies to treat LCH, whether to regard it as an autoimmune disorder or as a malignancy [11]. Though the literature hypothesizes neoplastic pathogenesis for LCH, the literature also describes autoimmune pathogenesis involving a cytokine imbalance of IL-2, Il-6, and T-helper-2 cells. Though rare, LCH has been associated with other autoimmune processes, including Evans syndrome and systemic lupus erythematosus [13]. Further research is needed to determine specific molecular mechanisms in the pathogenesis of LCH in order to help identify targets for therapeutic options, and subsequent *in vitro* and *in vivo* studies examining therapeutic responses to targeted therapy.

Treatment options for LCH are quite complex but primarily involve steroid and chemotherapy regimens. Research on the treatment of LCH is still ongoing with multiple large-scale prospective trials taking place. Prior to initiation of treatment, patients are risk-stratified, with liver, spleen, or bone marrow involvement portending a higher risk and a worse prognosis [14]. Moreover, it is also important to stratify patients into single-organ versus multisystem LCH. Treatment options generally include a single agent such as prednisone or a combination of a chemotherapy agent such as vinblastine with prednisone. Other options include resection or topical therapy for localized skin lesions [1,10,14]. Furthermore, studies have facilitated the use of BRAF inhibitors such as Vemurafenib into the therapies used for LCH after discovering the BRAF V600 mutation [9].

Autoimmune hepatitis is a chronic disorder that can present acutely and is characterized by persistent hepatocellular necrosis and inflammation [3]. According to European studies, the incidence of AIH can be up to 2 per 100,000 population per year, making it relatively much more common than LCH [4-5]. The disease is known to affect females more than males with up to 10:1 predominance in some studies [4-6]. AIH has no ethnic predilection and can present at any age within the population [6,7]. Similar to LCH, patients with AIH can present with symptoms such as fatigue, weight loss, abdominal pain, nausea, and itching. The patient presentation varies but can include clinical signs of cirrhosis or acute liver failure [7,15]. Though there is much uncertainty, the general hypothesis for the pathogenesis of AIH is that in genetically susceptible patients, an environmental stressor triggers a cascade of T-cell mediated events against self-antigens of the liver, eventually leading to progressive inflammation and AIH [7,15].

Although a diagnosis of exclusion, many laboratory and histological findings can help support a diagnosis of AIH. AIH is classified into two types, Type 1 and Type 2. Type 1, which is more common and occurs in middle-aged to elderly individuals, is characterized by the presence of ANA, ASMA, anti-soluble liver antigen/liver-pancreas antigen antibodies (anti-SLA/LP), and, infrequently, anti-mitochondrial antibodies (AMA) [11,15]. Type 2 AIH, observed more commonly in children and teenagers, is characterized by anti-liver kidney microsome-1 antibodies (anti-LKM1) and anti-liver cytosol-1 antibodies (ACL1). Elevated liver enzymes can be found in both types of AIH [11,15]. In Type 1 AIH, ANA are the most common circulating autoantibodies and are sometimes the only autoantibody found, whereas ASMA is less prevalent but more specific for AIH [4]. The autoantibody profile in this patient was compatible with a diagnosis of Type 1 AIH.

Generally, a serum ALT level at least five times the upper limit of normal values, serum immunoglobulin G (IgG) levels at least two times the upper limit of normal values or a positive ASMA, and a liver biopsy showing moderate or severe periportal or periseptal lymphocytic piecemeal necrosis is diagnostic of AIH [16-17]. A scoring system proposed by the International Autoimmune Hepatitis Group has provided specific characteristics that have been used to diagnose AIH. These characteristics include female sex, ALP/AST ratio, IgG levels, and autoantibodies such as ANA, and ASMA [16-17]. Using similar criteria, Hennes et al. proposed another diagnostic tool called the simplified AIH score, where patients who scored > 6 points were likely to have AIH [16]. More-specific criteria, named the Paris criteria, was made due to the complexity of diagnosing AIH in cases of overlap syndromes with primary biliary cirrhosis (PBC). Features of AIH are sometimes found in patients with PBC, such as elevated IGG, and the presence of ASMA. Moreover, patients with AIH may have features of PBC such as biliary abnormalities, and the presence of anti-mitochondrial antibodies (AMA) [17]. Studies still need to be done to further validate and compare such diagnostic criteria.

The patient’s liver biopsy helped support the diagnosis of AIH, as it showed severe acute hepatitis with a marked mixed portal and lobular inflammatory infiltrate consisting of numerous eosinophils and plasma cells, as well as pericellular, periportal, and sinusoidal fibrosis, with focal bridging consistent with AIH (Figure 2). According to the simplified AIH score, the patient’s high ANA titers, the presence of ASMA, IgG levels greater than 1.1 times the upper limit of normal, liver biopsy consistent with AIH, and absence of viral hepatitis point to AIH as being the likely cause of acute liver injury [16].

Treatment of AIH revolves around glucocorticoid monotherapy, however, combination therapies with azathioprine have been used. Although comparison studies are limited, clinical experience and the limited data illustrate that both treatment regimens are equally efficacious [18]. For this reason, the patient was started on a high-dose steroid regimen with the rationale that it would treat both LCH and AIH simultaneously. The patient's LFTs and rash began to improve with the steroid regimen, however, his hospital course was complicated by two episodes of lower GI bleed, the first of which the source was found and embolized, the second in which the source could not be found, necessitating a hemicolectomy.

Limited research exists regarding arterial gastrointestinal bleeds in patients with AIH or LCH. Most literature involving GI bleeds in AIH involves a degree of cirrhosis and portal hypertension leading to variceal bleeding. At this time, it is difficult to pinpoint what caused this patient's arterial GI bleed and whether his concomitant AIH and LCH contributed to the bleed. Specimen analysis from the hemicolectomy demonstrated atypical histiocyte proliferation positive for fascin, CD4, CD68, and CD1a. Although negative for stains such as S-100, the specimens had features similar to the lymph node and may have been signs of early involvement of systemic LCH in the colon. It is presumed that the systemic inflammatory state, underlying coagulopathy secondary to acute liver failure, episodes of mechanical ventilation, and high-dose corticosteroid therapy administered for the treatment of AIH and LCH increased the risk of GI bleeding in this patient. These severe physiologic stressors may have led to decreased mucosal blood flow and ischemia and subsequent formation of stress ulcers, as mechanical ventilation and coagulopathy are independent risk factors for stress ulcer formation [19]. A systematic review and meta-analysis examining corticosteroid use and the risk of GI bleeding concluded that corticosteroid therapy may increase the odds ratio for GI bleeding by up to 40% in hospitalized patients, as well as increasing the risk of bowel perforation [19]. 

## Conclusions

This case demonstrates a unique association of both LCH and AIH clinically presenting simultaneously. Given the extremely rare incidence of LCH, a medical coincidence of two rare disease processes occurring independently seems unlikely. As mentioned earlier, AIH is usually associated with a trigger in genetically susceptible patients. Given that this patient had LCH, it could have possibly triggered his AIH. Moreover, the inflammatory state of his AIH may have contributed to the proliferation of his LCH through tumorigenesis pathways. We also postulate that LCH may have some underlying autoimmune pathway that would make it susceptible to occur with other autoimmune disorders such as AIH. This case certainly contributes to the medical literature for these diseases and stimulates ideas for further research on the mechanism in which these rare diseases are interrelated, including the possibility that LCH has autoimmune pathways involved in its pathogenesis.
